# Dr. Henry S. Chase

**Published:** 1898-04

**Authors:** 


### Obituary.

### DR. HENRY S. CHASE.

Died at St. Louis, Mo., January 11, 1898, Henry S. Chase, M.D., D.D.S.

Dr. Chase was born at Saxon's Village, Vt., March 6, 1820. He graduated from the Medical College at Woodstock, Vt., in 1843. He subsequently attended the Baltimore Dental College, but did not graduate, the honorary degree of D.D.S. being, in after-years, conferred by Cincinnati Dental College.

He began the practice of dentistry in Woodstock, and resided there until 1856, when he moved to Iowa. He established himself in Iowa City, but in 1867 he accepted the offer of a chair in the Missouri Dental College at St. Louis.

Dr. Chase was a writer of marked ability and had made his name well known prior to his adoption of the somewhat celebrated "New Departure" creed. This radical separation from recognized methods of work made his name, together with his colleagues, a household word in dentistry.

The history of that movement, however mistaken it may appear in the light of the present, was a most interesting chapter in dental history in this country, with which the name of Chase must be associated with his still more pronounced associate, Dr. Flagg, of Philadelphia. That these views have had their influence cannot be denied, and that they have largely contributed to modify practice must be conceded, but whether this has been for the good or ill of the dental profession remains yet to be discovered.

Dr. Chase leaves a widow, four sons, and a daughter.

